# The Dominance of Anticipatory Prefrontal Activity in Uncued Sensory–Motor Tasks

**DOI:** 10.3390/s22176559

**Published:** 2022-08-31

**Authors:** Merve Aydin, Anna Laura Carpenelli, Stefania Lucia, Francesco Di Russo

**Affiliations:** 1 Department of Movement, Human and Health Sciences, University of Rome “Foro Italico”, 00135 Rome, Italy; 2 Department of Psychology, University of Rome “La Sapienza”, 00185 Rome, Italy; 3 Santa Lucia Foundation IRCCS, 00179 Rome, Italy

**Keywords:** ERP, CNV, pN, task preparation

## Abstract

Anticipatory event-related potentials (ERPs) precede upcoming events such as stimuli or actions. These ERPs are usually obtained in cued sensory–motor tasks employing a warning stimulus that precedes a probe stimulus as in the contingent negative variation (CNV) paradigms. The CNV wave has been widely studied, from clinical to brain–computer interface (BCI) applications, and has been shown to emerge in medial frontoparietal areas, localized in the cingulate and supplementary motor areas. Several dated studies also suggest the existence of a prefrontal CNV, although this component was not confirmed by later studies due to the contamination of ocular artifacts. Another lesser-known anticipatory ERP is the prefrontal negativity (pN) that precedes the uncued probe stimuli in discriminative response tasks and has been localized in the inferior frontal gyrus. This study aimed to characterize the pN by comparing it with the CNV in cued and uncued tasks and test if the pN could be associated with event preparation, temporal preparation, or both. To achieve these aims, high-density electroencephalographic recording and advanced ERP analysis controlling for ocular activity were obtained in 25 volunteers who performed 4 different visuomotor tasks. Our results showed that the pN amplitude was largest in the condition requiring both time and event preparation, medium in the condition requiring event preparation only, and smallest in the condition requiring temporal preparation only. We concluded that the prefrontal CNV could be associated with the pN, and this activity emerges in complex tasks requiring the anticipation of both the category and timing of the upcoming stimulus. The proposed method can be useful in BCI studies investigating the endogenous neural signatures triggered by different sensorimotor paradigms.

## 1. Introduction

Anticipatory brain functions are fundamental to keeping up with the rapid and continuous changes in the external world. Anticipation allows for speeding up the cognitive processes required to perform everyday tasks, from crossing the street to competing in sports.

Anticipation has often been investigated by means of electroencephalography (EEG) because this technique allows measuring, with millisecond precision, the brain activity preceding any sensorial or motor event. Using event-related potentials (ERPs), many studies individuated a series of slow potentials initiating up to several seconds before sensory or motor events. One of the most studied anticipatory potentials is the Bereitschaftspotential (BP) or readiness potential (RP), which precedes any voluntary movement and has been associated with motor preparation intended as the progressive excitability of premotor brain areas and behavioral readiness for forthcoming movements [1,2]. Another important anticipatory brain potential is the contingent negative variation (CNV), which is defined as the slow negativity arising between a warning stimulus defined as S1 or cue, and an imperative stimulus defined as S2 or probe [3]. The early centrofrontal phase of the CNV has been associated with orienting and expectancy, while the late phase has been associated with motor preparation and is identified with BP [4]. The early CNV was localized in the thalamus and the anterior cingulate and insular cortex, while the late CNV was identified in the cingulate and supplementary motor areas [5,6].

More recently, another anticipatory negativity has been described in sensory–motor tasks requiring stimulus discrimination. The prefrontal negativity (pN) initiates around one second before the onset of an uncued imperative stimulus requiring discrimination and possible response emission [7]. The pN peaks at stimulus onset and is maximal over prefrontal sites; it has also been associated with top–down cognitive preparation in the inferior frontal gyrus [8] mainly controlling attentional and inhibitory processing [9,10,11].

Anticipatory prefrontal activity has been known since 1970 [12], later labeled as stimulus-preceding negativity (SPN) by Damen and Brunia [13]. However, the SPN precedes feedback stimuli conveying task performance information [14] and non-imperative stimuli requiring response emission such as the oddball and the go/no-go tasks. Before the study of Berchicci et al. [7], prefrontal activity anticipating imperative stimuli was hardly reported, likely because of its small amplitude and because the contamination of ocular artifacts in recordings of frontal ERP has always been a significant measurement problem. Despite this, some early ERP studies reported the possible existence of prefrontal CNV [15,16], which was confirmed by Weinberg and Papakostopoulos [17] in a study controlling eye movements. In these studies, the prefrontal CNV was medial and smaller than the central and parietal CNV, and it had no hemispheric prevalence depending on the responding hand. These early studies only differentiated this frontal activity from the standard parietal CNV, and no attempts to explain its functional significance were made. For almost 50 years, no other studies were aimed to establish the nature of this CNV. With the help of modern ocular artifact removal methods such as those based on independent component analysis (ICA), the prefrontal anticipatory activity has been investigated in many studies and is associated with response accuracy (the larger the pN, the higher the accuracy). Considering that the BP has been associated with response speed (the larger the BP, the faster the response time), the pN and BP have been defined as the neural basis of proactive speed/accuracy trade-off and have been proposed to reflect a proactive accelerator/brake system that, based on predictive internal models, makes plans and anticipates future actions [8,18].

A feature of the pN still not defined is whether it represents an event or temporal preparation [19]. Event preparation is characterized by uncertainty about which one of several alternative actions is going to be executed. Temporal preparation comes into play if it is uncertain when to execute the action.

Furthermore, research on anticipatory ERP such as the pN may be important in other domains such as novel training techniques for rehabilitation, education, and daily activities using brain–computer interfaces (BCIs). EEG-based BCI relies mostly on reactive activity (consequent to stimulations) such as the P3 ERP or steady-state evoked potentials. Since in BCI, there is a need to understand the user’s intention, anticipatory ERP should be more suited than reactive activity. A recent study [20] showed that the EEG activity during time prediction can be used in active BCI controls. However, the CNV was often overlooked due to measurement difficulties in classifying the complex CNV structure [21]. In general, the signals that can be used for active BCI are still underdeveloped and lack variety [20].

The aim of this study was to confirm the existence of the prefrontal CNV using state-of-the-art ERP analyses and to define its functional significance by identifying possible similarities or dissimilarities with the pN found in uncued discrimination tasks. In addition, we aimed to characterize this prefrontal activity testing to better understand whether it denotes event preparation or temporal preparation, or both. Finally, considering that an open question in active BCI investigation is which kind of cognitive function can express the brain’s intention in a more direct way, the present study investigated the endogenous neural signatures triggered by different sensorimotor paradigms and tested the separability of corresponding anticipatory ERP signatures.

To achieve these aims, we compared the anticipatory ERP activity during the fore period before the onset of a probe stimulus (S2) in tasks differing by the presence of S1 cues (uncued vs. cued conditions) and differing by the information conveyed by the cue. This information was either temporal only (indicating the imminent arrival of probe stimuli requiring a discriminative response; time-cued condition), or both temporal and event information (indicating, in addition, whether the probe stimulus will require a response or not; target-cued and non-target-cued conditions). Even though some investigations showed the presence of a prefrontal CNV, none were sure of its authenticity because of the ocular artifacts problem, and it was never associated with the prefrontal anticipatory activity found in uncued tasks. If confirmed, this association may help to increase our knowledge of anticipatory prefrontal functions, expanding the possible EEG-based active BCI applications.

According to the literature, we expect that the prefrontal CNV will be larger in tasks requiring a discriminative response. Moreover, if the pN is associated with temporal preparation, it should be larger in uncued than in cued tasks. Otherwise, if it is associated with event preparation, it should be larger in a task with S1 not informing about the required response than in a task with S1 probe stimuli indicating the required responses. If the pN is associated with both temporal and event preparation, we expect a gradient in the three mentioned task conditions. Finally, if the pN (as the BP) is strictly related to anticipation in tasks requiring a motor response, it should be absent in tasks with S1 probe stimuli indicating that no response is required. The BP is not the focus of this study but should be identical in all tasks requiring responses and absent in the task not requiring them. In addition, the BP slope should be steeper in a task requiring a discriminate response than in simple response tasks [9].

## 2. Materials and Methods

### 2.1. Participants

The sample size was determined with the G*power 3.1.9.2 software [22], estimating effect size from Cohen’s f statistics. We set a medium expected effect size f for the present repeated-measure 4-level ANOVA design at 0.25; the α level was set at 0.05, and the desired power (1 − β) at 0.83 (estimated minimum sample size of 25). Therefore, 25 young adult university students (12 females, 13 males, mean age 27.0 years; SD = 7.1) were recruited for the study. The inclusion criteria were the following: absence of any neurological and psychiatric disorders, absence of any medication during the experimental sessions, normal or corrected-to-normal vision, being fully right-handed (Edinburgh handedness inventory [23]), and naive about the aim of the study. All participants gave their informed consent before participating in this study in accordance with the Declaration of Helsinki after approval by the local ethical committee of the University of Rome “Foro Italico”.

### 2.2. Procedure

Four visuomotor discriminative response tasks (DRTs) (i.e., the go/no-go paradigm) were conducted during EEG recordings in the Cognition and Action Neuroscience Laboratory at the University of Rome “Foro Italico”. Participants underwent the EEG montage, afterward being asked to sit in front of a computer screen that was 114 cm away from their eyes with their right-hand palm down on a push-button board. During the experiment, a black background with a yellow fixation point (0.15 × 0.15° in diameter) was visible in the center of the screen in a dimly lit, quiet room. The stimulus-onset asynchrony differed between 3 and 4 s for the four visual stimuli (250 ms duration with equal probability (*p* = 0.25)), which were square shapes measuring 4 × 4° and made up of either vertical or horizontal bars. This was introduced to avoid ERP overlaps with the preceding and subsequent stimuli. The experimenter stressed both speed and accuracy, instructing participants to press the button as soon as they could only when (two out of four) predetermined target stimuli appeared on the screen (*p* = 0.5) and to withhold the motor response when non-target stimuli occurred (*p* = 0.5). Between runs, the four stimuli were randomized. Each run lasted for two minutes with a break in between. Delivering 32 runs, we were able to complete 1280 trials in 64–70 min. 

Differences among the four conditions were the following:

1. Uncued: This basic task was exactly as described above. With the appearance of only the probe (target or non-target) stimuli;
2. Time-cued: In addition to the basic task, a 250 ms cue stimulus (purple fixation point) appeared 1500 ms before the probe stimulus. This cue only informed about the probe stimulus arrival in 1500 ms;
3. Target-cued: In addition to the basic task, a 250 ms cue stimulus (green fixation point) appeared 1500 ms before the probe stimulus. This cue informed the observer that the upcoming probe stimulus category will be a target;
4. Non-target-cued: In addition to the basic task, a 250 ms cue stimulus (red fixation point) appeared 1500 ms before the probe stimulus. This cue informed the observer that the upcoming probe stimulus category will be a non-target.

**Figure 1:**
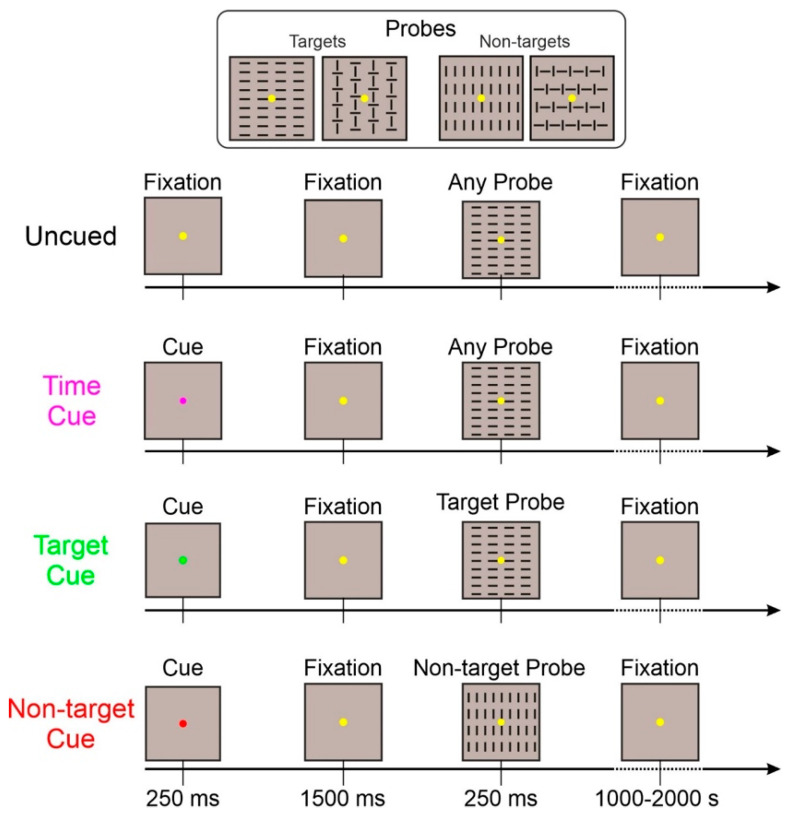
Representation of the four task conditions. The fixation point is larger than the original to emphasize its color. In the uncued and time-cued conditions, any of the four probes could be displayed. In the target-cued condition, only the two target probes could appear. In the non-target-cued condition, only the two non-target probes could appear.

Target- and non-target-cued tasks were randomized within the same run; the other two tasks were administered in separate runs. The sequence of the three kinds of runs was randomized. The same number of trials was collected for each task. A schematic representation of the four tasks is displayed in Figure 1.

### 2.3. Behavioral Data

Median response times (RTs) for correct trials were calculated for each participant. Accuracy was calculated as a percentage of omissions (OM, i.e., missed responses to target stimuli), and commission errors (false alarm (FA), i.e., erroneous responses to non-target stimuli).

### 2.4. EEG Recording

EEG was continuously recorded using the Recorder 1.2 software and three BrainAmp^TM^ amplifiers, two of which were connected to 64 active sensors ActiCap; data were processed using the Analyzer 2.2.2 software (all by BrainProducts GmbH, Munich, Germany). Electrodes were mounted according to the 10-10 international system and referenced to the M1 electrode. EEG data were amplified, digitized at 250 Hz, band-pass-filtered using a Butterworth zero-phase filter (0.01–40 Hz and 50 Hz notch filter; second-order), and stored for offline analyses. Eye movements were monitored with an electrooculogram (EOG) recorded by the third BrainAmp amplifier (ExG type) in bipolar modality. Horizontal EOG was recorded with an electrode pair over the left and right outer canthi of the eyes, while vertical EOG (VEOG) was recorded with an electrode pair below and above the left eye. Electrode impedances were kept below 5 KΩ. After referencing, the EEG to the M1–M2 average, blink, and vertical eye movement artifacts were automatically corrected by means of the independent component analysis. Data were then submitted to automatic artifact rejection, excluding EEG with amplitudes exceeding the threshold of ±70 µV. About 2.4% of trials were rejected, with no significant difference between the conditions (F_(3,72)_ < 1).

To evaluate the prestimulus activity, EEG was segmented into 1900 ms epochs, starting 1600 ms before and ending 300 ms after the probe stimulus onset. The baseline was applied from −1600 to −1500 ms, 100 ms before the cue onset in the cued conditions. Given that, in the uncued and in the time-cued conditions, the knowledge of stimulus category was unpredictable at the prestimulus stage of processing, the target and non-target trials were averaged together. On other hand, given the full predictability of the probe category of the target- and non-target-cued conditions, each one included only the relative probe.

To select the intervals and electrodes to be considered in statistical analysis, the “collapsed localizer” method was used [24] in which a localizer ERP is obtained by collapsing (averaging) all experimental conditions. To identify the interval of analysis, the global field power (GFP) was calculated. The GFP describes the ERP spatial variability at each time point considering all scalp electrodes simultaneously, resulting in a reference-independent descriptor of the potential field. The prestimulus interval in which the GFP was larger than 80% of its maximum value was used for further analysis. This GFP approach selected one interval from −800 ms to 0 ms in which the mean amplitude was calculated in all conditions for statistical purposes. The electrodes with an amplitude larger than 80% of the maximum value in the intervals selected by the collapsed localizer were joined in spatial pools and considered for statistical analysis. Two foci of activity were clearly present: the prefrontal activity of the pN and the centroparietal activity of the BP components. The pN was then represented by a pool containing Fp1, Fpz, Fp2, AF3, AFz, and AF4 electrodes (prefrontal pool). The BP was represented by a pool containing F1, Fz, F2, FC1, FCz, and FC2 electrodes (frontocentral pool). Voltage and current source density (CSD) mapping were used to show ERP scalp topography.

### 2.5. Statistical Analysis

For behavioral and EEG measures, the Shapiro–Wilk W test was performed to test the assumption of normality. The test showed non-significant values for all the considered measures, confirming their normal distributions. To test the assumption of homoscedasticity, the Levene test for equality of variance was performed, showing no violation of the sample homoscedasticity. Further, considering that distributions of RT are typically skewed to the right, we calculated skewness for these data obtaining a value close to zero, thus assuming that the distribution of RT data is approximately symmetric. After this preliminary testing, behavioral measures (RT, omissions, and false alarms) were submitted to ANOVA with one factor (condition) at three levels (uncued, time-cued, and target-cued). ERP measures (pN and BP) were submitted to ANOVA with one factor (condition) at four levels (uncued, time-cued, target-cued, and non-target-cued). Effect sizes were reported in terms of partial eta squared (ηp^2^). For post hoc comparisons, the Bonferroni correction was used. The overall alpha level was fixed at 0.05. All statistical analyses were performed using the Statistica 12.0 software (StatSoft Inc., Tulsa, OK, USA).

## 3. Results

### 3.1. Behavioral Data

**Figure 2:**
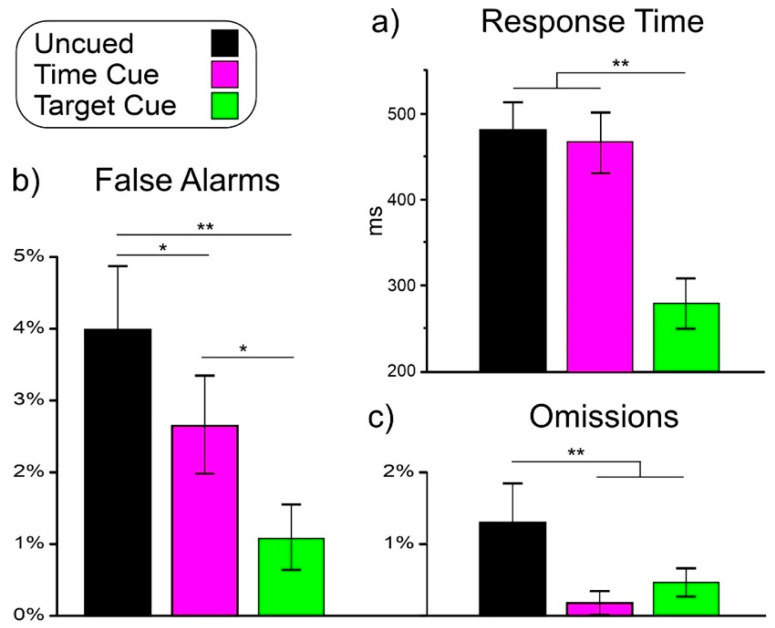
Behavioral results in the three tasks: (**a**) response time; (**b**) false alarms; (**c**) omissions. Vertical bars denote 0.95 confidence intervals. * *p* < 0.05, ** *p* < 0.01.

Figure 2 shows the behavioral results in the uncued, time-cued, and target-cued conditions only because no responses were emitted in the non-target-cued condition.

#### 3.1.1. Reaction Time

ANOVA on RT showed a significant effect of condition (F_(2,48)_ = 123.1, *p* < 0.001, ηp^2^ = 0.837). Post hoc comparisons showed that RT in the target-cued condition (289 ms) was lower (*p* < 0.001) than in the other two conditions, which did not differ from each other (uncued: 480 ms, time-cued: 466 ms).

#### 3.1.2. False Alarm

ANOVA on false alarms showed a significant effect of condition (F_(2,48)_ = 10.3, *p* < 0.001, ηp^2^ = 0.300). Post hoc comparisons showed that the false alarm percentage in the uncued condition (4.02%) was higher (*p* < 0.021) than in the other two conditions. The false alarm percentage in the time-cued condition (2.66%) was higher (*p* < 0.017) than in the target-cued condition (1.04%).

#### 3.1.3. Omission

ANOVA on omissions showed a significant effect of condition (F_(2,48)_ = 6.6, *p* = 0.003, ηp^2^ = 0.217). Post hoc comparisons showed that the omission rate in the uncued condition (1.31%) was higher (*p* < 0.003) than in the other two conditions, which did not differ from each other (time-cued: 0.19%, target-cued: 0.44%).

### 3.2. ERP Results

**Figure 3:**
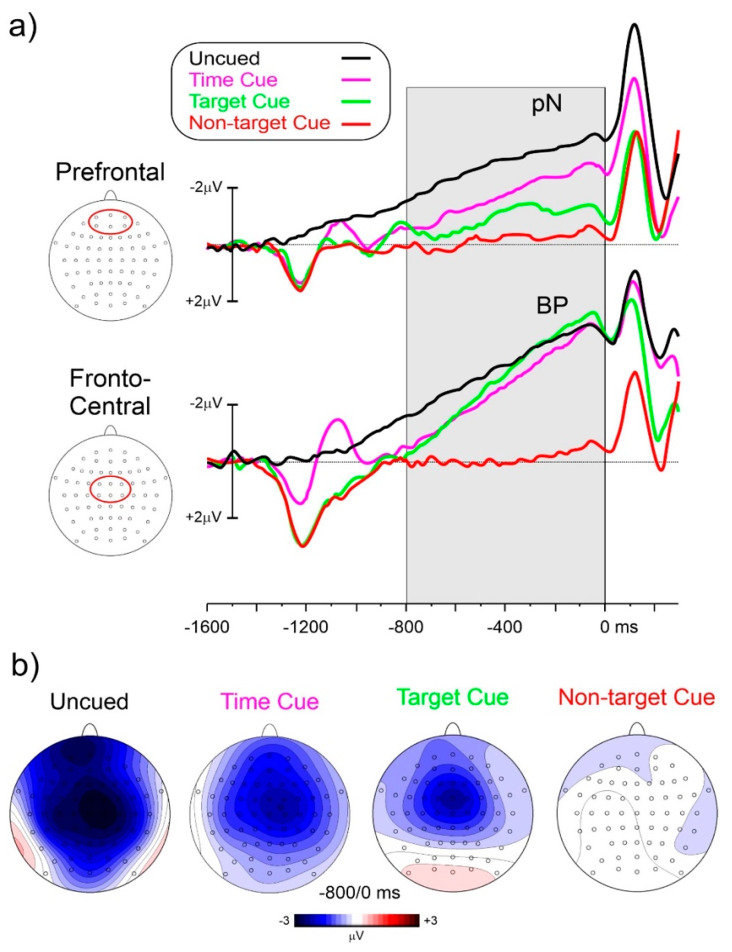
(**a**) ERP waveforms at the medial prefrontal and frontocentral pools of electrodes, which are indicated with a red shape in the head flat-view insets. The grey area indicated the studied interval; (**b**) scalp topography in the window from −800 to 0 ms showing the prefrontal pN and the frontocentral BP from top-flat views for the studied conditions.

Figure 3a shows the prestimulus ERP waveforms for the four experimental conditions. Figure 3b shows the topographical voltage distribution from −800 to 0 ms. The cue onset produced a positive deflection peak at 280 ms at the medial centroparietal sites, and in the time-cued condition, a further negative peak over parietal areas. However, the focus of interest was the later period within 800 ms before the probe stimulus onset. In these windows, both the prefrontal (pN) and frontocentral negative activities were present with different amplitudes depending on the conditions. In the uncued condition, the pN was much earlier, initiating at about 1250 ms before the probe stimulus onset. The BP initiated at about −1150 ms. In the other conditions, the pN onset was at about −900 ms, and the BP onset was at about −850 ms. In the non-target-cued condition, flat prestimulus waveforms were detected in the −800/0 ms interval.

**Figure 4:**
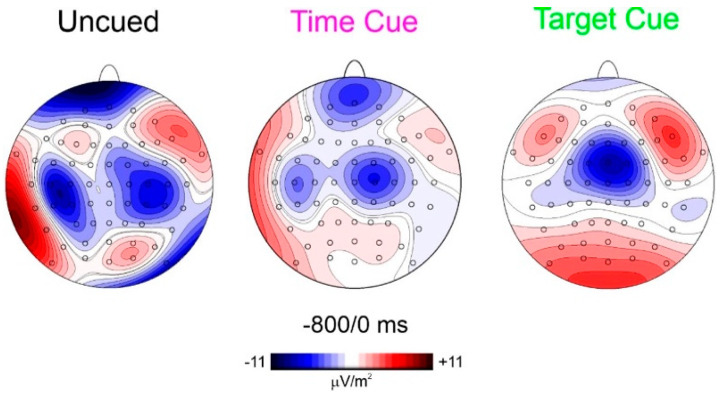
CSD scalp distribution in the window from −800 to 0 ms shows the presence of prefrontal activity with different gradients.

To best characterize the spatial features of the found anticipatory ERP, CSD mapping was also employed (Figure 4) showing the prefrontal foci of activity in all three conditions but with different magnitudes.

ANOVA on pN showed a significant effect of condition (F_(3,72)_ = 31.2, *p* < 0.001, ηp^2^ = 0.567). Post hoc comparisons showed that pN amplitude in the uncued condition (−2.65 μV) was larger (*p* < 0.003) than in the other conditions. The pN amplitude in the time-cued condition (−1.51 μV) was larger (*p* = 0.025) than the target-cued (−0.99 μV) and the non-target-cued (−0.06 μV, *p* < 0.001) conditions. The pN amplitude in the target-cued condition was larger (*p* < 0.001) than in the non-target-cued one.

ANOVA on the BP showed a significant effect of condition (F_(3,72)_ = 60.0, *p* < 0.001, ηp^2^ = 0.471). Post hoc comparisons showed that the BP amplitude in the non-target-cued condition (−0.08 μV) was smaller (*p* < 0.002) than in the other conditions, which did not differ from each other (uncued: −3.58 μV, time-cued: −3.20 μV, target-cued: −3.36 μV).

**Figure 5:**
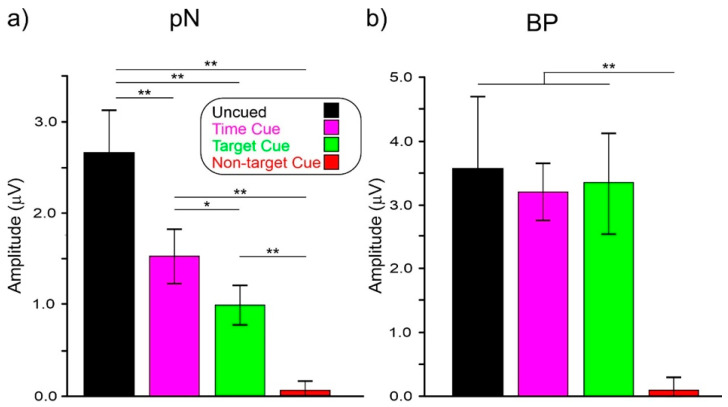
(**a**) Amplitudes of the pN and BP; (**b**) components in the −800/0 ms interval in the four conditions. Vertical bars denote 0.95 confidence intervals. * *p* < 0.05, ** *p* < 0.01.

Figure 5a graphically shows the pN amplitudes and Figure 5b, the BP amplitudes in the studied conditions.

## 4. Discussion

In this study, we compared the brain potentials preceding imperative probe stimuli in two CNV and one non-CNV paradigm, differing by the presence of S1 cue stimuli and by the information conveyed by cues. The aim was to confirm the existence of a prefrontal CNV and to possibly explain its functional significance by associating it with the prefrontal negativity (pN) found in uncued discriminative response tasks. In addition, we aimed to better characterize the pN by testing its possible association with event preparation, temporal preparation, or both.

At the behavioral level, the response time was similar and slow in the uncued and time-cued tasks (about 470 ms), while in the target-cued task, it was much faster (290 ms), thus associating this condition with a simple response task [9]. Regarding accuracy, the most challenging task was the uncued followed by the time-cued and the target-cued tasks.

At the electrophysiological level, the non-target-cued condition produced no prestimulus negativities while in both the time-cued and target-cued conditions the CNVs initiated about 900 ms before the S2. In the uncued condition, negativities initiated earlier (at about −1200 ms) and were larger than in other conditions at prefrontal sites. At frontocentral sites, the amplitudes were comparable.

Regarding the first aim of the study, relating to prefrontal CNV, in both the time-cued and target-cued tasks, the CNV was dominant in frontocentral areas as in the literature [25], but a smaller prefrontal focus was recognizable in the target-cued condition and is clearly detectable in the time-cued condition, as evident by CSD mapping (Figure 4). This result confirms the early CNV literature [15,16,17] finding prefrontal contribution to CNV. In addition, the more recent literature using subdural recordings is also confirmed [26]. Considering that the most popular CNV paradigm resembles the present time-cued condition and the concern of confusing eye movements related to the prefrontal activity, the activity of using an uncued discrimination response task (similar to the present uncued task) in a population (old adults) that presented prefrontal hyperactivity and, therefore, very large anticipatory prefrontal negativity, remained substantially unconsidered until Berchicci [7]. Since the found similarities, the prefrontal CNV may be assimilated with the pN and, therefore, considered the neural basis of top–down anticipatory cognitive control for the upcoming task. The results show that this control is high in response tasks requiring both temporal and event preparation (uncued condition), medium in response tasks requiring only event preparation (time-cued condition), minimal in response tasks not requiring discrimination (target-cued condition), and absent in tasks not requiring response at all (non-target-cued condition).

Regarding the second aim related to the prefrontal CNV and the pN functional significance, the results showing that the pN dominated in the uncued condition indicate that this component subtends both event and temporal preparation indexed by increasing amplitude. The similar topography of the prefrontal CNV and the pN suggests that the two activities may be assimilated, and both may index top–down cognitive functions, mainly controlling attentional and inhibitory processing.

The prefrontal CNV and pN components depend on the upcoming task, showing a larger amplitude gradient than the parietal CNV and BP. Therefore, it may be possible to predict the upcoming subject action, helping the information transfer rate, in order to test the EEG-based BCI. In addition, it has been shown that CNV-like activities are well-suited for single-trial detection in active BCI paradigms even using imaginary responses [27]. In light of the findings, it could be interesting to replicate our study without requiring motor functions and instead rely upon the intention to move during different (cued and uncued) scenarios. This would allow BCI paradigms with a low cognitive load, making a more natural brain–computer interaction possible, as suggested by recent studies [28]. In this regard, in EEG-based BCI systems for motor imagery tasks, a novel methodology such as the adaptive empirical wavelet transform (EWT)-based signal decomposition might be used for classification accuracy since its application demonstrated significant achievement [29]. Regarding the difficulty of being accurate with the imagery state of each subject, which can vary dramatically depending on the task and the volunteer’s mental state, EWT may help with analyzing the most significant modes for each channel. Additionally, in future studies, components such as visual–motor awareness, stimulus-response mapping, and decision-making aspects may contribute to the design and advancement of EEG-based BCI systems as well as to anticipatory prefrontal activity. To avoid missing variable brain signals in such a circumstance of increased component complexity, measurements and analysis approaches may be enhanced or integrated using several classifications, such as EWT, ICA, and principal component analysis (PCA).

To summarize, we confirmed the existence of a prefrontal CNV component, which is assimilable to the pN found in uncued tasks. This component seems associated with both temporal and event preparation. This activity could be used in active BCI paradigms because the neural representation of an intention could develop more than a second prior to response emission [30].

## Data Availability

Data are available from the corresponding author upon request.
